# Locking Plates With Computationally Enhanced Screw Trajectories Provide Superior Biomechanical Fixation Stability of Complex Proximal Humerus Fractures

**DOI:** 10.3389/fbioe.2022.919721

**Published:** 2022-06-23

**Authors:** Dominic Mischler, Jana Felicitas Schader, Jan Dauwe, Lara Tenisch, Boyko Gueorguiev, Markus Windolf, Peter Varga

**Affiliations:** ^1^ AO Research Institute Davos, Davos, Switzerland; ^2^ Department of Trauma Surgery, UZ Leuven, Leuven, Belgium

**Keywords:** bone fracture (MeSH ID: D050723), osteosynthesis, implant optimization, 3D-printing, finite element analysis, fixation failure

## Abstract

Joint-preserving surgical treatment of complex unstable proximal humerus fractures remains challenging, with high failure rates even following state-of-the-art locked plating. Enhancement of implants could help improve outcomes. By overcoming limitations of conventional biomechanical testing, finite element (FE) analysis enables design optimization but requires stringent validation. This study aimed to computationally enhance the design of an existing locking plate to provide superior fixation stability and evaluate the benefit experimentally in a matched-pair fashion. Further aims were the evaluation of instrumentation accuracy and its potential influence on the specimen-specific predictive ability of FE. Screw trajectories of an existing commercial plate were adjusted to reduce the predicted cyclic cut-out failure risk and define the enhanced (EH) implant design based on results of a previous parametric FE study using 19 left proximal humerus models (Set A). Superiority of EH versus the original (OG) design was tested using nine pairs of human proximal humeri (*N* = 18, Set B). Specimen-specific CT-based virtual preoperative planning defined osteotomies replicating a complex 3-part fracture and fixation with a locking plate using six screws. Bone specimens were prepared, osteotomized and instrumented according to the preoperative plan *via* a standardized procedure utilizing 3D-printed guides. Cut-out failure of OG and EH implant designs was compared in paired groups with both FE analysis and cyclic biomechanical testing. The computationally enhanced implant configuration achieved significantly more cycles to cut-out failure compared to the standard OG design (*p* < 0.01), confirming the significantly lower peri-implant bone strain predicted by FE for the EH versus OG groups (*p* < 0.001). The magnitude of instrumentation inaccuracies was small but had a significant effect on the predicted failure risk (*p* < 0.01). The sample-specific FE predictions strongly correlated with the experimental results (R^2^ = 0.70) when incorporating instrumentation inaccuracies. These findings demonstrate the power and validity of FE simulations in improving implant designs towards superior fixation stability of proximal humerus fractures. Computational optimization could be performed involving further implant features and help decrease failure rates. The results underline the importance of accurate surgical execution of implant fixations and the need for high consistency in validation studies.

## 1 Introduction

Standardized fracture fixation implants provide good outcomes for most trauma applications. However, there are still problematic sites for osteosynthesis such as the proximal humerus. Besides intramedullary nailing, locking plate fixation has become one of the most commonly used joint-preserving surgical treatment options (Sudkamp et al., 2009). Nevertheless, even with state-of-the-art locked plating of proximal humerus fractures, the rate of mechanical fixation failures has been reported to range between 15% and 35% (Krappinger et al., 2011; Kralinger et al., 2014; Hengg et al., 2019; Panagiotopoulou et al., 2019), or even higher in the most endangered patient group of elderly osteoporotic women with complex and unstable fractures (Krappinger et al., 2011). The already high incidence is expected to further increase due to the aging population and prevalence of osteoporosis (Bahrs et al., 2014). Moreover, targeted studies reported no clear advantage of locked plating versus conservative treatment in terms of reoperation rate or functional outcomes (Olerud et al., 2011; Fjalestad et al., 2012; Rangan et al., 2015; Handoll et al., 2017). The potential reasons for mechanical fixation failures are manifold and include aspects related to the patient, complexity of the fracture, surgical execution, and the currently available implant designs that may not provide optimal fixation stability.

Conventional *in vitro* testing is the current gold standard method to evaluate biomechanical competence of implant fixations (Jabran et al., 2018), however, it is limited to the investigation of selected aspects, ideally in a paired study design, and therefore not well suited for analysis of complex multifactorial problems. Computational modeling enables rapid feedback on the implant stability under a wide variety of complicated conditions and can be used to investigate the effect of selected aspects in a systematic and efficient manner (Lewis et al., 2021). We have developed and validated a finite element (FE) simulation tool kit to analyze the virtual biomechanical behavior of locking plate fixations of proximal humerus fractures (Varga et al., 2017; Varga et al., 2018b). This “virtual testing machine” has been used to explore various aspects of locking plate fixations including the effects of configuration (Fletcher et al., 2019c), length (Fletcher et al., 2019a) and augmentation (Varga et al., 2020) of the locking screws, as well as positioning (Fletcher et al., 2019b) and type (Mischler et al., 2020a) of the plate. Beyond the analysis of existing implants, FE simulations can be utilized to explore the possibility of improving fixation stability by adapting design features. In recent *in silico* studies, the predicted cut-out failure risk could be significantly reduced by optimizing the screw trajectories of a fixed-angle locking plate (Mischler et al., 2020b) and further benefits were observed using patient-specific implants (Schader et al., 2021). The underlying FE analysis methodology was validated to predict experimental cyclic cut-out failure of locked plating of unstable three-part proximal humerus fractures (R^2^ = 0.90, *N* = 19) (Varga et al., 2017). However, the validation has been performed for a given implant design and configuration only, and it has not been demonstrated whether the design improvements suggested by the simulations would indeed result in superior biomechanical outcomes, or whether this would be outside the validity scope. Further, it was unclear how accurately the planned configuration could be implemented experimentally and how potential instrumentation inaccuracies would affect the outcomes.

Therefore, the primary aim of this study was to evaluate the biomechanical benefit of a locking plate with computationally enhanced screw trajectories versus the standard implant design in a paired human cadaveric study. The second aim was to evaluate instrumentation accuracy and its potential influence on the FE predictions. The third aim was to quantify the specimen-specific predictive ability of FE simulations of both the planned and instrumented states.

## 2 Materials and Methods

**FIGURE 1:**
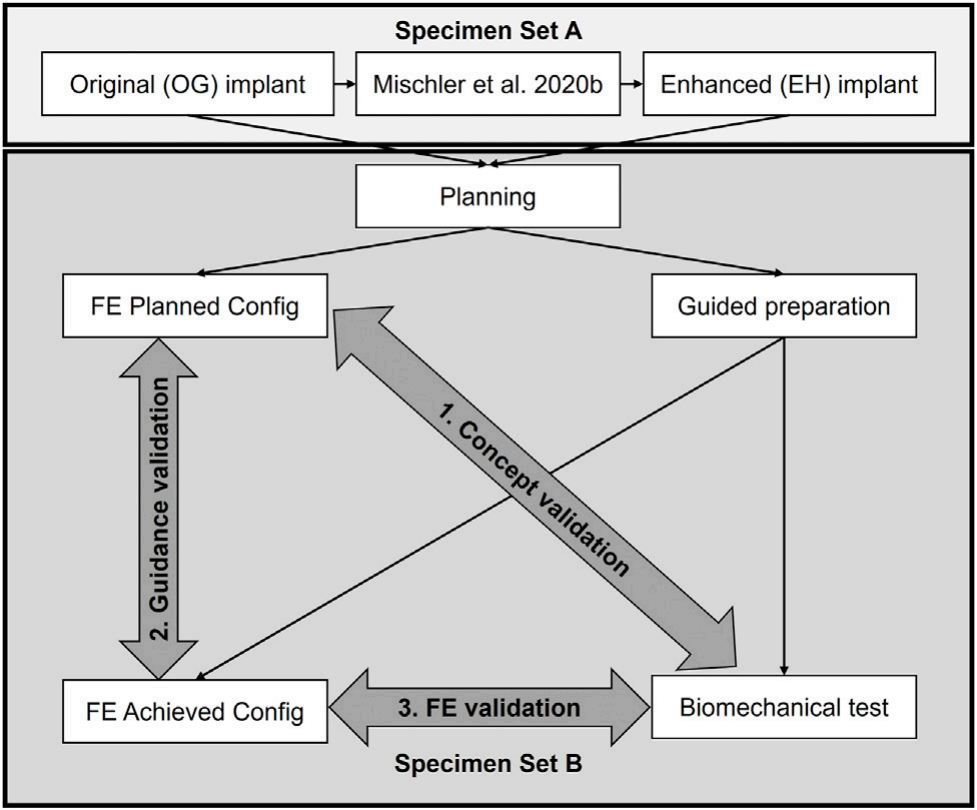
Study overview. A computationally enhanced (EH) screw trajectory configuration was defined based on the results of a previous finite element (FE) analysis study (Set A) and compared with the original (OG) design *via* biomechanical testing on human cadaveric humerus specimens (Set B) to validate the concept of in silico design improvement (aim 1). The accuracies of the guided instrumentation (aim 2) and the specimen specific FE predictions (aim 3) were also validated based on the data of Set B.

This study used a coupled computational and experimental approach, where FE simulations informed about how to enhance the implant configuration, and the biomechanical tests evaluated whether the updated implant was superior to its original design, in line with the primary aim (Figure 1). Finally, FE models of the experimentally achieved constructs were created to determine the effect of potential instrumentation inaccuracies (second aim) and the specimen-specific predictive power (third aim). This was achieved in five steps (Figure 1): 1) the trajectories of the locking screws were varied compared to the original (OG) implant design of the PHILOS plate (DePuy Synthes, Zuchwil, Switzerland) in a previous parametric FE simulation study on 19 digital proximal humerus specimens (Set A) and used to define the enhanced (EH) configuration by reducing the predicted failure risk; 2) 18 paired bone specimens with high intra-donor symmetry (Set B) were osteotomized and fixed with 3D-printed metal plates featuring the OG or the EH screw trajectory designs based on preoperative planning, assisted by subject-specific 3D-printed guides; 3) the cyclic cut-out failure of the OG and EH fixations were assessed *via* biomechanical testing and compared between the groups; 4) instrumentation accuracy was evaluated by comparing the planned and experimentally achieved configurations; 5) FE simulations of both planned (FE-Planned) and experimentally achieved (FE-Achieved) configurations were performed and compared with the experimental results *via* correlation analysis.

### 2.1 Finite Element-Based Definition of the Enhanced Implant Configuration

**FIGURE 2:**
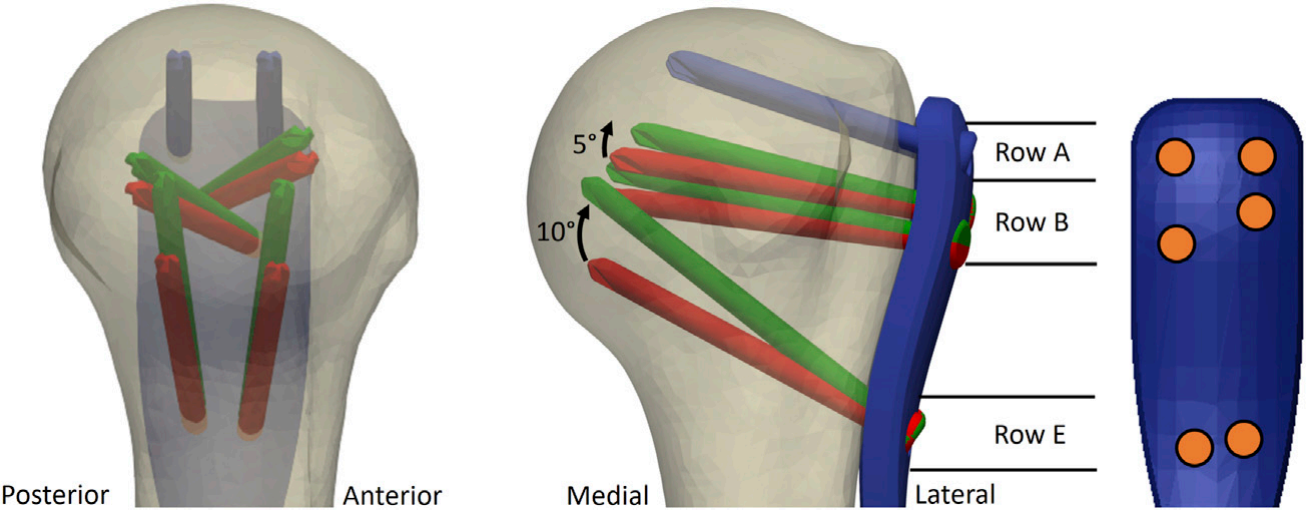
Illustration of the original (OG, red) and the enhanced (EH, green) screw trajectories used for the experimental validation study. The most proximal screw row (blue) was the same for both OG and EH designs. Screws of row E were rotated proximally by 10° and screws of row B by 5°, respectively, compared to the OG configuration. Note that the intact bone is used only for illustration; the fixation was optimized in virtual fracture models. The screw distribution on the plate is illustrated with orange circles.

The screw orientations of the EH implant design were defined based on the results of a previous parametric FE study (Mischler et al., 2020b). In short, FE models of nineteen low-density left proximal humerus specimens (Set A) of nine female and ten male elderly donors (mean ± standard deviation (SD) age: 83 ± 9 years) were created using a computational osteosynthesis tool (Varga et al., 2018b) to simulate an unstable three-part fracture AO/OTA 11-B3.2 instrumented with the PHILOS plate using six proximal locking screws occupying rows A, B and E (Figure 2). In a parametric analysis, the trajectories of these screws were individually varied in a grid-like pattern in both anterior-posterior and superior-inferior directions with 5° increments. Three physiologically relevant loading modes were applied. The average compressive principle strain in the cylindrical bone regions around the screws—a validated predictor for cyclic cut-out failure risk (Varga et al., 2017)—was evaluated for each new screw orientation configuration, compared to the OG design taken as baseline and finally averaged for all 19 samples.

These results were used in the current study to identify the combination of screw trajectories of the EH configuration that provided the largest overall observed decrease in the predicted cut-out risk compared with OG configuration while considering potential spatial restrictions of small humeral head sizes and anterior-posterior symmetry. The symmetry aspect was required for application of the same plate design to both left and right bones. Accordingly, compared with OG, the EH implant design was defined by proximally shifting the tips of screws in rows B and E by 5° and 10°, respectively, while keeping row A unchanged (Figure 2).

### 2.2 Specimen Selection for Set B

Human cadaveric humeri with low bone mineral density (BMD) and high pair symmetry (Set B) were identified from a larger pool in order to minimize the confounding effect of intra-donor differences. All donors gave their informed consent inherent within the donation of the anatomical gift statement during their lifetime. All experiments were carried out under the relevant guidelines and regulations. Additionally, internal review boards at Science Care (Phoenix, AZ, United States) and AO Research Institute Davos (Davos, Switzerland) approved the project. Thirty fresh-frozen (−20°C) humeral pairs from elderly donors were scanned using computed tomography (CT, GE Revolution, GE Healthcare, United States) with scanning settings of 120 kV voltage, 200 mA current and 0.625 mm slice thickness. The Hounsfield unit values of the CT image voxels were converted to BMD using a density calibration phantom (QRM-BDC/6, QRM GmbH, Moehrendorf, Germany). Humeri with high BMD (> 130 mgHA/cm^3^) were excluded from the study. Radius and BMD of the humeral head were evaluated using previously developed methods (Varga et al., 2018a). In order to avoid confounding intra-donor differences, pair symmetry was evaluated in terms of humeral head radius (difference < 3%) and BMD (difference < 10%). Additionally, the humeral heads were required to be large enough to accommodate the calcar screws (Row E, Figure 2) of the OG configuration. The corresponding evaluation was performed on anterior-posterior C-arm images of the intact bones using an in-house developed implant navigation system (Windolf and Richards, 2021). Nine pairs (donor age: 85.3 ± 5.2 years, range: 73–91 years) fulfilled all criteria and were selected and scanned *via* high-resolution peripheral computed tomography (HR-pQCT, XtremeCT, Scanco Medical AG, Brüttisellen, Switzerland) with 60 kVp voltage, 900 µA current and 82 µM isotropic voxel size.

### 2.3 Preoperative Planning and Finite Element Simulations

The osteotomies and implant fixations were planned for each specimen of Set B based on the HR-pQCT images using Amira software (v2019, Thermo Fisher Scientific, Hillsboro, OR, United States) to maximize consistency and pair symmetry, thereby decreasing the effect of potential confounding factors. The detailed description of the planning procedure can be found in the Supplementary Material.

**FIGURE 3:**
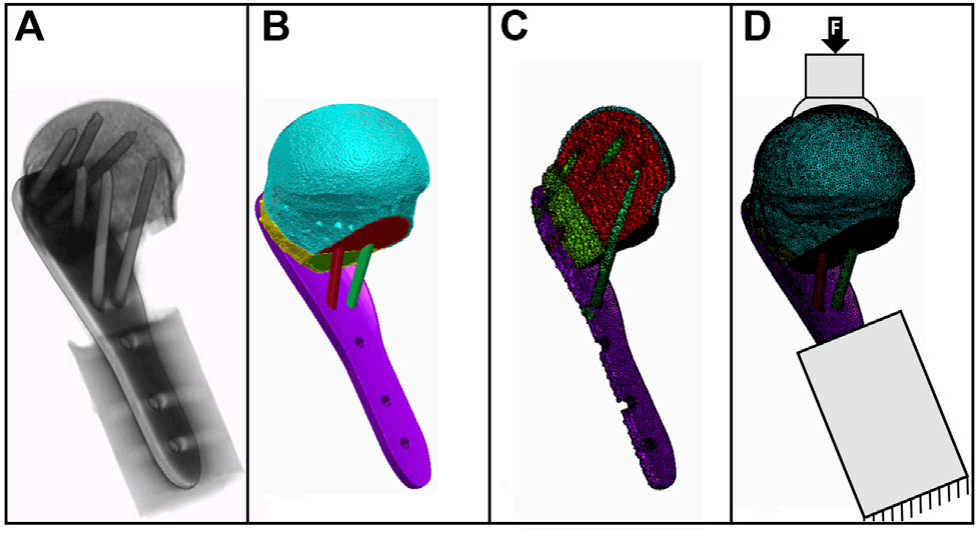
Gray value image of a postoperative CT scan **(A)** with the underlying mask of bone fragments and implants **(B)**; the cross-sectional view of the FE mesh **(C)** and the specimen-specific FE model with boundary conditions are illustrated as fixed at the distal part and with load applied on the bone surface **(D)**.

Specimen-specific FE models were created based on the planned osteotomy and implantation settings (Figure 3) using an established workflow (Varga et al., 2018b). Using Simpleware M-2017.06 (Simpleware Ltd., Exeter, United Kingdom), the bone fragment domains taken from the virtually osteotomized HR-pQCT images were combined with the computer-aided design (CAD) models of the plate and screws. The cortical and trabecular bone regions were separated using a special fill algorithm using Medtool v3.8 (Dr. Pahr Ingenieurs e.U., Pfaffstätten, Austria) (Medtool, 2014). The models were meshed with linear tetrahedral elements of 0.3–1.0 mm edge length. All material properties were isotropic and linear elastic. The screws and the 3D-printed metal plate (see Section 2.4) were modelled as made of titanium (modulus of elasticity 105 GPa) and steel (modulus of elasticity 210 GPa), respectively (Synthes, 2009). The elastic properties of the bone elements were scaled from the HR-pQCT-based BMD values (Dragomir-Daescu et al., 2011). Plate-screw and screw-bone interfaces were modeled as bonded. The FE models were aligned, and the boundary conditions were set according to the loading case of the planned experimental setup (section 2.6). The embedded portion of the plate was fully constrained and a static vertical force of 100 N was applied in a distributed manner on the proximal part of the humeral head (Figure 3). The FE analyses were performed using Abaqus (v2019, Simulia, Dassault Systemes, Velizy-Villacoublay, France). Average peri-implant strain was evaluated in cylindrical regions of the trabecular bone around the screws with a diameter of 8 mm and a total length of 50 mm, starting 5 mm in front of the screw tips (Varga et al., 2017).

Both OG and the EH configurations were simulated for each specimen. The two specimens of each pair were then stratified in the two groups based on the FE simulation results to avoid potential grouping bias.

### 2.4 Implant Manufacturing

Eighteen proximal humerus locking plates were fabricated with powder-based additive manufacturing *via* direct metal laser sintering from steel (1.2709, powder size of 15–45 µM) using an M2 device (CONCEPT Laser GmbH, GE Additive, Lichtenfels, Germany) at BSF Bünter AG (Heerbrugg, Switzerland). Printing settings were laser power of 200 W, layer thickness of 30 µM and thermal treatment at 540°C for 8 h to remove residual stresses and increase yield strength and hardness. The shape of the plates mimicked the PHILOS design with an increased wall thickness of 4 mm to eliminate potential plate bending during biomechanical testing. The screw holes with conical threads for the 3.5 mm locking screws were subsequently tapped by CNC machining. Trajectories of the six proximal screws followed the OG and EH designs for all nine plates in each group.

### 2.5 Specimen Preparation and Instrumentation Using 3D-Printed Guides

**FIGURE 4:**
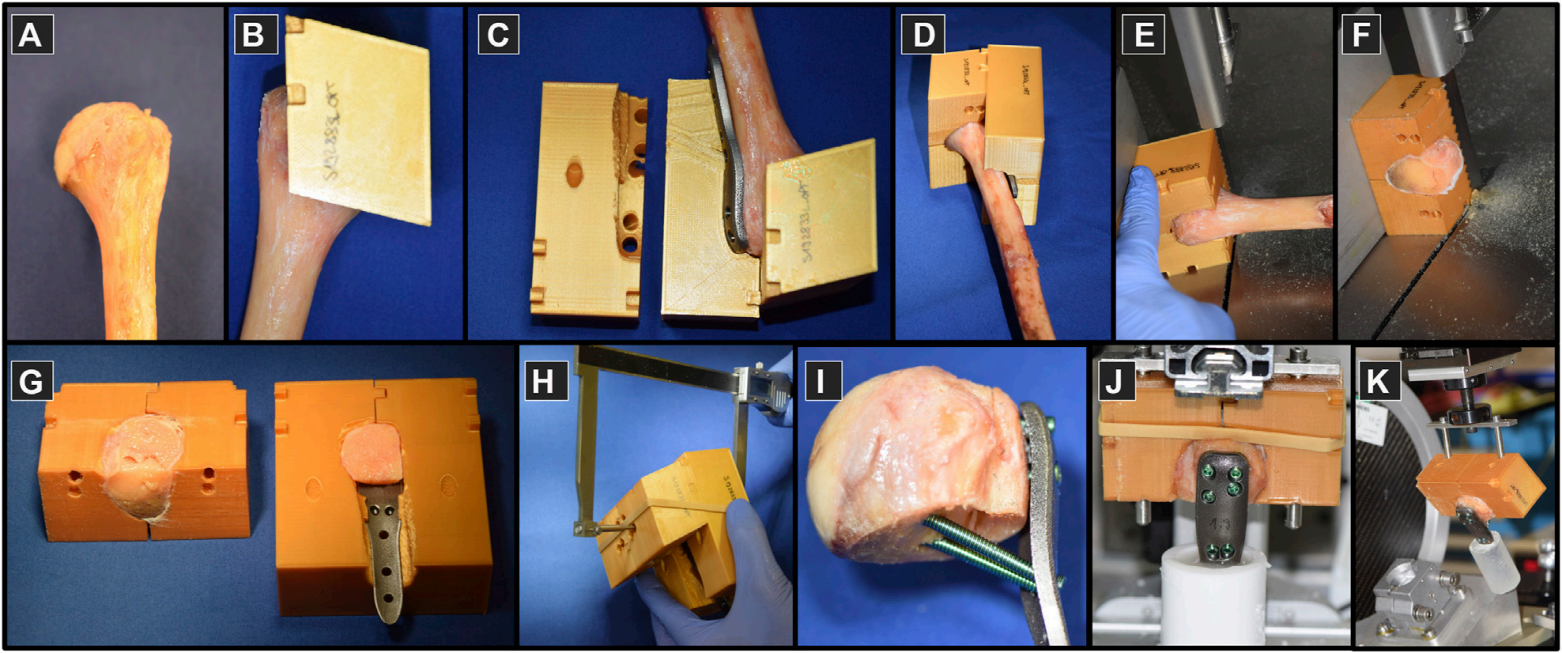
Specimen preparation on proximal humeri **(A)** using custom 3D-printed plastic guides **(B–D)** used to assist the process of osteotomizing **(E,F)**, fragment reduction and implant positioning **(G)**, instrumentation **(H,I)**, embedding **(J)** and mounting to the experimental setup for biomechanical testing **(K)**.

Individualized guides were designed and manufactured to aid the experimental execution of the planned specimen preparation procedures, including osteotomies, fragment reduction, implant positioning, pilot hole drilling, screw insertion, alignment of embedding and mounting on the testing machine (Figure 4). The guides were designed using a custom-developed semi-automated workflow in Amira (Supplementary Material) and 3D-printed from polylactide (Ultimaker 3, Ultimaker B.V., Utrecht, Netherlands).

Prior to processing, the bones were thawed at room temperature and purged of soft tissue. The fit of the humerus and the plate into the guides was then tested (Figures 4A–D). The surgical neck was osteotomized first using a bandsaw with blade thickness of 0.4 mm (Figure 4E). The second plane on the guide was used to cut the lateral fragment with the greater tuberosity from the main head fragment (Figure 4F). The bone fragments were reduced and the construct was assembled, including the 3D-printed plate (Figure 4G). For each screw, the required drill depth and screw length were controlled using a custom caliper to provide approximately 6 mm tip-to-joint distance, considering the 2 mm increments of the commercial screws (Figure 4H). The pilot holes were drilled using the standard drill sleeves of the PHILOS instrument set. The fixation was instrumented by occupying all six holes of rows A, B and E with 3.5 mm titanium locking screws (DePuy Synthes, Zuchwil, Switzerland) (Figure 4I) using standard screw sleeves aligned by the guiding block holes. The distal part of the plates was embedded direct distally to the calcar screws in Polymethylmethacrylate (PMMA, SCS-Beracryl, Suter-Kunststoffe AG, Fraubrunnen, Switzerland) with the guide utilized to align the construct along the humerus anatomical shaft axis (Figure 4J). In the final step, the guide ensured correct alignment of the specimen with respect to the loading axis of the biomechanical test setup while mounting it to the machine base (Figure 4K). Each specimen was CT-scanned after plating (post-operative scan) using the same device and settings as described above. Using these CT images, the deviation of the achieved instrumentation compared to the planned configuration was evaluated in terms of screw angles and tip-to-joint distances (Supplementary Material).

### 2.6 Biomechanical Testing

**FIGURE 5:**
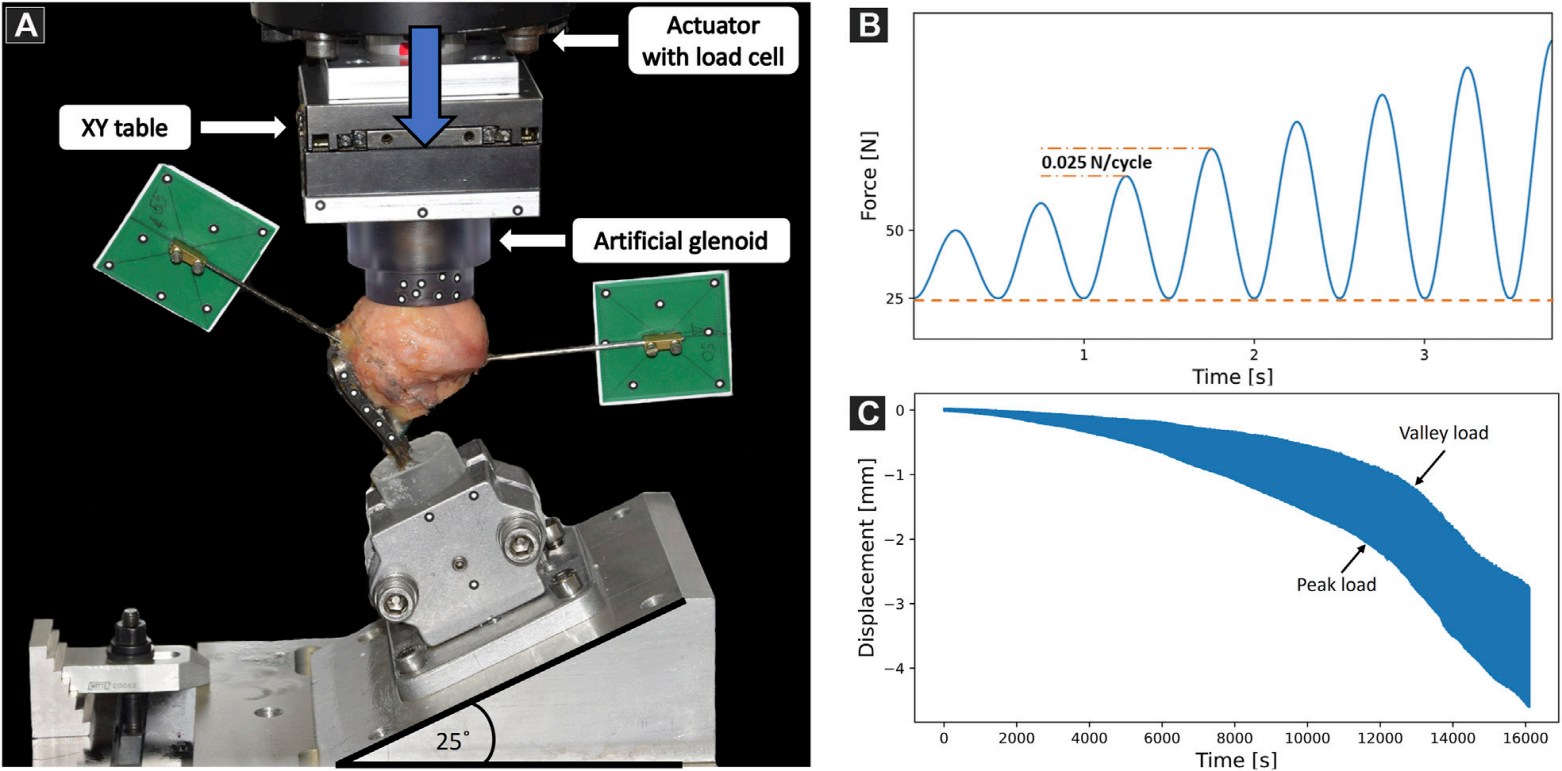
**(A)** Biomechanical test setup with a specimen mounted on a metal wedge, inclined at 25° with respect to the machine base. Vertical arrow indicates loading direction. Motion tracking markers were attached to track the artificial glenoid, the plate, the mounting jig and the two bone fragments. **(B)** Biomechanical loading protocol consisting of sinusoidal curves with constant valley load (dashed line) and gradually increasing peak load (dashed-dotted line). **(C)** Exemplary results showing the evolution of humeral head center displacement along the anatomical shaft axis over time.

The setup for biomechanical testing was designed to provide a physiologically relevant loading mode of the proximal humerus and to ensure reproducibility according to the FE models. The bone-implant constructs were mounted to the machine base in a lateral angulation of 25° to replicate a physiologically relevant loading regime (Bergmann et al., 2011). To alleviate shear forces acting on the humeral head, a horizontal sliding table was attached to the machine actuator, allowing the artificial glenoid cup to continuously center itself on the humeral head during mechanical testing (Figure 5A). The specimens were cyclically tested to failure using an electrodynamic material testing machine (Acumen, MTS, Eden Prairie, MN, United States). The test protocol consisted of a sinusoidal loading curve with a constant valley load of 25 N and a gradually increasing peak load at a rate of 0.025 N/cycle, starting at 50 N (Figure 5B). The test was stopped at 6 mm actuator displacement. Stereographic motion tracking was used to measure the translational and rotational degrees of freedom of each component of both the setup and fixation construct using Aramis SRX camera system (GOM GmbH, Braunschweig, Germany). The head center was determined by fitting a sphere through digitized points on the head surface assessed with a touch probe device. The main parameter of interest was the displacement of the humeral head center along the humeral shaft axis relative to the plate (Figure 5C). Cut-out failure was defined as residual head fragment displacement at valley load; various failure threshold levels were evaluated, ranging from 0.25 mm to 1.5 mm with steps of 0.25 mm. The primary outcome measure of the experimental biomechanical testing was the number of cycles to cut-out failure.

### 2.7 Finite Element Modeling of the Experimentally Achieved Constructs

Subject-specific FE models of the plated specimens were created based on the postoperative CT scans to consider the experimentally achieved osteotomies, fracture reduction, implant positioning, screw orientation and embedding. The domain and position of the bone fragments were evaluated on the postoperative CT image in Amira. To avoid the effect of metal artefacts—present in the postoperative CT scan—on the BMD-based material properties, the image regions of the bone fragments were replaced by the spatially co-registered HR-pQCT image of the intact humerus. The experimentally achieved positioning of the plate and screws was replicated based on the postoperative scan by registering the CAD surfaces of the implants. The PMMA embedding of the distal part of the plate, determining specimen alignment and boundary conditions of the experimental test setup, was analyzed by fitting a cylinder to the embedding region in the postoperative scan. The FE-Achieved models of each specimen were then created and peri-implant strain was evaluated using the same simulation methodology as described in section 2.3.

### 2.8 Statistical Analysis

Standard statistical methods were used for general data analysis using the SciPy package (Virtanen et al., 2020) in Python programming language (v3.7.4, Python Software Foundation, https://www.python.org/). Normality of data distribution was checked with Shapiro-Wilk test. Biomechanical stability was assessed by the number of cycles to cut-out failure and compared between the OG and EH groups by means of paired two-sided t-test or two-sided Wilcoxon Signed-Rank test in case of normally or non-normally distributed data, respectively. Linear regression analysis was performed and Pearson’s correlation coefficient was computed to evaluate the strength of the relationship between the experimental number of cycles to failure and the peri-implant strain. Level of significance was set to 0.05 for all statistical tests.

## 3 Results

### 3.1 Specimen Characteristics in the Study Groups

No significant differences were observed between the two groups of Set B in terms of BMD (OG mean ± SD: 112.0 ± 15.5 mgHA/cm^3^, EH mean ± SD: 112.2 ± 13.7 mgHA/cm^3^, *p* > 0.94, *N* = 9 pairs) or head radius (OG: median: 21.5 mm, range: 20.6–23.7 mm; EH: median: 21.5 mm, range: 20.6–23.5 mm; *p* = 0.07, *N* = 9 pairs).

### 3.2 Concept Validation: Comparison Between Original and Enhanced

FE results of the FE-Planned configurations demonstrated that average peri-screw bone strain was significantly lower for the EH (mean ± SD: 367 ± 52 µmm/mm, range: 285–441 µmm/mm) versus the OG (mean ± SD: 460 ± 44 µmm/mm, range: 387–515 µmm/mm) implant designs, *p* < 0.001, *N* = 9 pairs.

**FIGURE 6:**
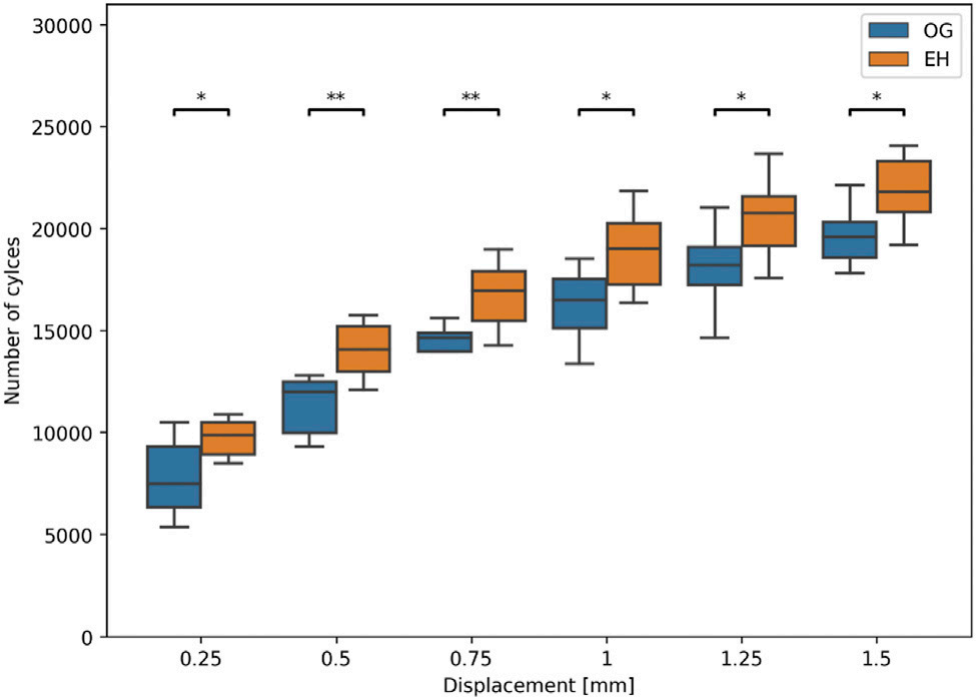
Experimental number of cycles to cut-out failure at different levels of residual relative head fragment displacement with respect to the plate. Asterisks indicate significant differences between OG (blue) and EH (orange) implant designs. (*: *p* ≤ 0.05; **: *p* ≤ 0.01).

During biomechanical testing, one pair was excluded due to issues with motion tracking data acquisition. Significantly higher cycles to failure were observed in the EH versus OG group for all levels of residual head fragment displacement, with the strongest significance being at 0.5 mm threshold (OG: mean ± SD: 11′368 ± 1′313 cycles, range: 9′320–12′814 cycles; EH: mean ± SD: 14′080 ± 1′414 cycles, range: 12′097–15′773 cycles), *p* = 0.01, *N* = 8 pairs (Figure 6).

### 3.3 Guidance Validation: Comparison Between Planned and Achieved Configurations

The deviation of the experimentally achieved screw trajectories compared with the planned state was 0.3 ± 1.3° (mean ± SD; range: −2.5–3.9°) in the proximal-distal direction and −1.7 ± 1.8° (range: −5.5–4.7°) in anterior-posterior directions. tip-to-joint distance deviations were −0.3 ± 1.1 mm (range: −3.1–2.2 mm). Detailed results are provided in the Supplementary Material. However, when replicating the instrumented state of the specimens with the FE simulations, significantly higher peri-screw bone strains were found for the FE-Achieved (mean ± SD: 514 ± 89 µmm/mm, range: 343–654 µmm/mm) compared with the FE-Planned analyses (mean ± SD: 414 ± 67 µmm/mm, range: 285–516 µmm/mm), p < 0.01, *N* = 18 specimens.

**FIGURE 7:**
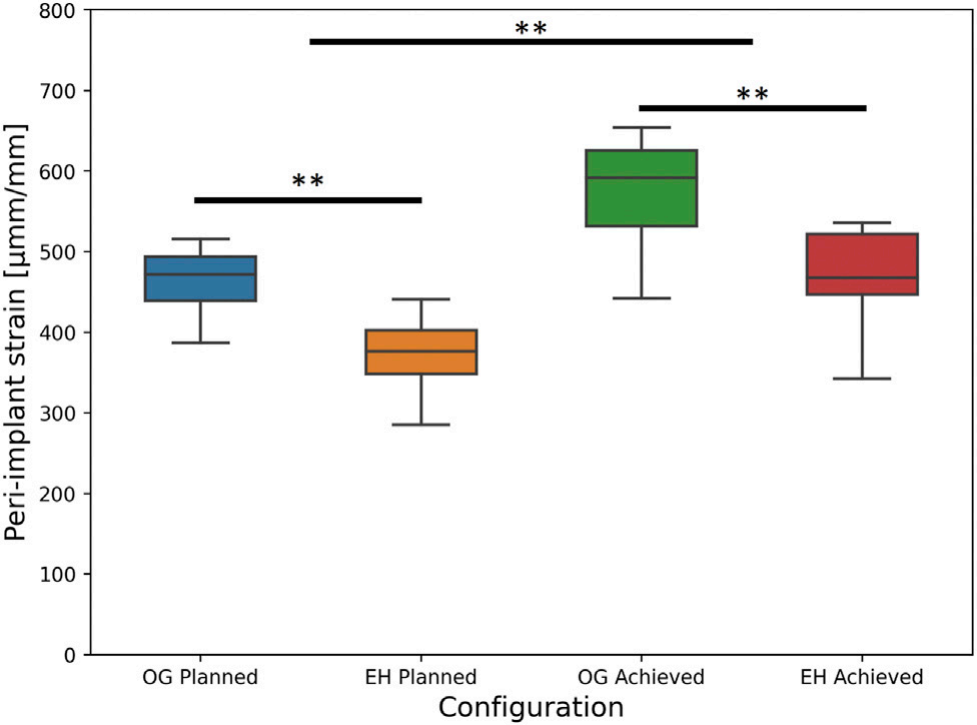
FE-based peri-implant bone strain results, i.e., predicted risk of cut-out failure, for the planned (left) and achieved (right) model types, with the latter exhibiting significantly higher values versus the former; significant differences between the OG and EH screw configurations are demonstrated within each separate (planned or achieved) model type (**: *p* ≤ 0.01).

Nevertheless, the imperfections appeared to have similar influence on both groups and did not affect the statistical findings regarding the difference between the plate designs. In line with the FE-Planned simulations, the FE-Achieved models provided significantly lower peri-screw bone strains for the EH implant group (mean ± SD: 461 ± 71 µmm/mm, range: 343–536 µmm/mm) compared with the OG group (mean ± SD: 568 ± 73 µmm/mm, range: 441–654 µmm/mm), p = 0.01, *N* = 9 pairs (Figure 7).

### 3.4 Finite Element Validation Against Biomechanical Results

**FIGURE 8:**
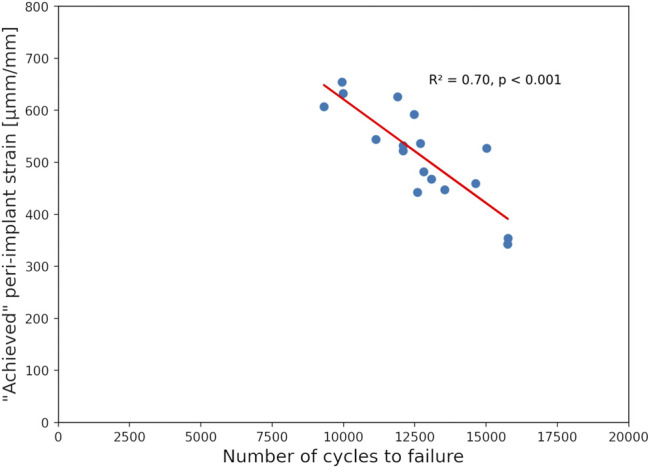
Correlation of the experimental number of cycles until 0.5 mm cut-out failure and the peri-implant strain of the achieved FE models showing good prediction accuracy with high significance.

No significant correlations were found between the experimental biomechanical results and the FE-Planned simulations. In turn, results of the FE-Achieved models demonstrated a good correlation with the number of cycles to cut-out failure, providing R^2^ = 0.70 at the 0.5 mm failure threshold (*p* < 0.001, *N* = 17 specimens, Figure 8).

## 4 Discussion

Conventional validation studies aim to demonstrate that the computer models can reproduce previously observed experimental results. The present study took the more challenging inverse route to investigate whether the design changes suggested by the simulations would lead to experimentally measurable improvements. Indeed, this work demonstrated the feasibility and validity of the *in silico* approach for implant improvement as the computationally enhanced locking plate design provided significant increase in the biomechanically assessed number of cycles to cut-out failure versus the standard design in a complex unstable proximal humerus fracture model. This was in line with the significantly lower peri-implant bone strain observed for the EH versus OG design in the FE simulations.

Improving primary stability of proximal humerus fracture fixations is not straightforward. Although the failure of single screws is strongly determined by the density of the surrounding bone region (Hepp et al., 2003; Steiner et al., 2015; Panagiotopoulou et al., 2021), FE simulations can provide improved predictions of stability for screws (Panagiotopoulou et al., 2021) and especially for entire bone-implant constructs (Varga et al., 2018a). FE analyses can incorporate the complex effects related to implants fixed with several screws, such as the importance of screw spread (Fletcher et al., 2019c). These benefits render computational tools optimal for investigation of implant improvement options in parametric analyses. Previous studies by Jabran et al. and Mischler et al. proposed FE-based optimization of screw trajectories of proximal humerus locking plates; however, without any biomechanical corroboration using human cadaveric specimens (Jabran et al., 2019; Mischler et al., 2020b). The present work filled this gap by demonstrating that an FE analysis methodology previously developed and validated to predict cyclic failure of a given implant design (Varga et al., 2017) remains valid to indicate design changes towards improved primary stability. This underlines the power and potential of validated computational methods that could be utilized towards tackling the challenging problem of fracture fixation failures.

The major change in the enhanced design providing biomechanical benefit found here was the elevation of the screw tips, i.e., proximal rotation of the screw trajectories. This is in line with the previous *in silico* optimization studies (Jabran et al., 2019; Mischler et al., 2020b). The present work used the same implant design for left and right humeri, in analogy with the PHILOS plate. Accordingly, the changes in screw trajectories compared with the original design were kept symmetric. Side-specific implants may achieve higher benefits and subject-specific designs may further improve performance (Schader et al., 2021). These were not considered in this study due to logistical challenges. Some commercially available implants feature side-specific designs to achieve benefits in fit and purchase, however, would require double stock to be kept available at hospitals. Towards subject-specificity, some plates utilize variable-angle locking designs, but even for these, the screw orientations providing optimal fixation stability remain unknown. Several studies have investigated the biomechanical behavior of locking plates with polyaxial screw holes, allowing a screw angulation range of 30–40° (Erhardt et al., 2009; Ruchholtz et al., 2011; Voigt et al., 2011; Zettl et al., 2011; Erhardt et al., 2012). However, the orientation of the screws within the humeral head were chosen by the surgeon during instrumentation based on intuition and thus the highest stability was potentially not achieved. This might be the reason why no superiority to fixed-angle locking plates could be demonstrated to date (Ockert et al., 2010; Voigt et al., 2011; Katthagen et al., 2016). The freedom of versatile configurations provided by these implants may not ensure the best stability. The selection of the case-specific screw arrangement and orientation of these implants can be arbitrary; it may not be reliable, reproducible, and mechanically optimal. Methodologies such as the FE analysis presented in this study, once developed further to the level of clinical application and high automation, could help in subject-specific screw trajectory selection.

Although reaching significance, the relative gain of the adjusted implant configuration compared with the original design remained moderate, approximately 19%. Further research could utilize the validated FE workflow for more generic optimization broadened to other features of the implant design. In future, this computer simulation methodology could be transferred to *in vivo* applications, to evaluate subject-specific fracture stability preoperatively for various implant choices and configurations, and guide the surgeon about the best possible individualized treatment option for the given patient. Nevertheless, biomechanical studies always represent an idealized scenario, while the clinical reality is more complex. Generally, biomechanical studies use osteotomies and anatomically correct fracture reductions. In clinics, the fracture pattern is usually more complex, and the planned reduction may not always be achieved, thus altering the construct behavior and making *in silico* optimizations more challenging. Further clinical validation would be required to evaluate whether the enhanced implant design would ensure lower clinical failure rates.

Besides the implant design, the quality and surgical accuracy of execution is of high importance. The small inaccuracies of instrumentation revealed by the postoperative analysis indicated that the specimen-specific 3D-printed guides were efficient. However, even these relatively small imperfections had a considerable effect on stability, as revealed by the FE analysis of the achieved state, demonstrating a significantly higher predicted failure risk compared to the planned configuration. While the statistical finding of the FE-Planned and FE-Achieved analyses was the same concerning the superiority of the EH versus the OG design, the simulations revealed similar results for the OG-Planned and EH-Achieved groups, indicating that the benefit of trajectory improvement was comparable to the loss of experimental imperfections. These findings emphasize the relevance of intraoperative navigation and guidance that could help surgeons to accurately execute the preoperative plan (Windolf and Richards, 2021). Such technologies are expected to help lowering complication rates of proximal humerus fracture fixations.

Another implication of the instrumentation inaccuracies was the lack of correlation between the FE-Planned simulations and the biomechanical results. However, when incorporating the instrumentation inaccuracies in the models, the FE-Achieved simulations were able to predict the cycles of cut-out failure with a good accuracy (R^2^ = 0.70), confirming the validity of the used modeling approach in predicting biomechanical cut-out failure risk. These findings demonstrate the importance of incorporating exact details, including imperfections of the instrumentation, into FE modeling when attempting to predict results of biomechanical testing. This is particularly important for studies aiming at validation of computer simulations.

Several limitations should be considered in this study which extend beyond the general limitations of biomechanical human cadaveric studies. The bone density of the specimens may not have reflected the most endangered highly osteoporotic population, but the range was reasonable compared with previous biomechanical studies. The complex physiological loading conditions of the shoulder joint could not be replicated experimentally, but the used test setup incorporated the most important aspect, i.e., the direction of glenohumeral loads acting *in vivo* (Bergmann et al., 2007; Bergmann et al., 2011). Furthermore, the used loading mode was designed to ensure its replication in the boundary conditions of the FE simulations. Several simplifications were used in the FE model. The bone-screw interfaces were defined as fully bonded and the properties of all materials were isotropic and linear elastic. However, the same simplifications were used in the previous validation study that confirmed that the FE simulations could well predict experimental cyclic cut-out failure and found no improvements with more sophisticated bone-screw interface models (Varga et al., 2017). The validated outcome measure, i.e., bone strain around the screw tips, can be computed with linear elastic models and does not require more sophisticated description of the material behavior of bone. The used osteotomy model mimicked a single and idealized fracture pattern, but it represented a challenging complex unstable three-part fracture including a comminuted calcar region. The 3D-printed guides are not feasible in a clinical setting and only intended for biomechanical studies. Finally, the fixation failures observed in the clinics are often of multifactorial nature. The failure mechanism investigated in the present study was screw cut-out and thus the results may not be applicable to other failure modes such as screw perforation.

To conclude, this study demonstrated that computationally enhanced screw trajectories in locking plates could reach significantly higher number of cycles to cut-out failure compared to the original implant design during biomechanical testing of unstable proximal humerus fractures. These findings confirmed the validity of the FE-based improvement approach and reinforced the power of computational simulations. The presented computational approach could be extended to other features of the design and help decrease the rate of fixation failures with improved implants, although clinical validation would be required first. Instrumentation of the planned configurations was achieved with good accuracy using the custom guides, but even the slight imperfections had a significant effect on the predicted failure risk. This underlines the importance of accuracy in surgical execution and implant placement that can be a more dominant factor than the implant design, potentially absorbing the benefit of optimization, and may require intraoperative navigation to achieve optimal outcomes. The FE models could predict the specimen-specific biomechanical results only when replicating the experimentally achieved construct including the inaccuracies, indicating the need for reproducing exact details in validation studies.

## Data Availability

The raw data supporting the conclusions of this article will be made available by the authors, without undue reservation.
