# Supplementary material for: Locking Plates With Computationally Enhanced Screw Trajectories Provide Superior Biomechanical Fixation Stability of Complex Proximal Humerus Fractures
Source: Front Bioeng Biotechnol. 2022 Jun 23;10:919721. doi: 10.3389/fbioe.2022.919721 (PMC9260250; doi:10.3389/fbioe.2022.919721)
Supplement: Supplementary file 1 [file DataSheet1.DOCX]

Supplementary Material

# Specimen-specific planning – methodological details

The external bone contour was segmented on the high-resolution peripheral computer tomography (HR-pQCT) images and meshed with a triangular surface grid. For each pair, the model of the right humerus was mirrored and spatially registered to the contralateral left side. Osteotomy planes were defined to create unstable three-part fractures simulating a comminuted calcar region. The exact same osteotomy cut planes were then imposed on both bones of the pair and mirrored back to the original right humerus. For consistent implant positioning, the left and co-registered mirrored right bone geometries were merged into one bone volume. The plate was then positioned on the merged bone using a previously developed plate fitting algorithm (Varga et al., 2018b) supervised by a medical surgeon (Figure S1). The use of the merged model was necessary as, due to intra-subject asymmetries, plate fitting to one side only could have led to potential interference with the geometrical variations of the contralateral bone.


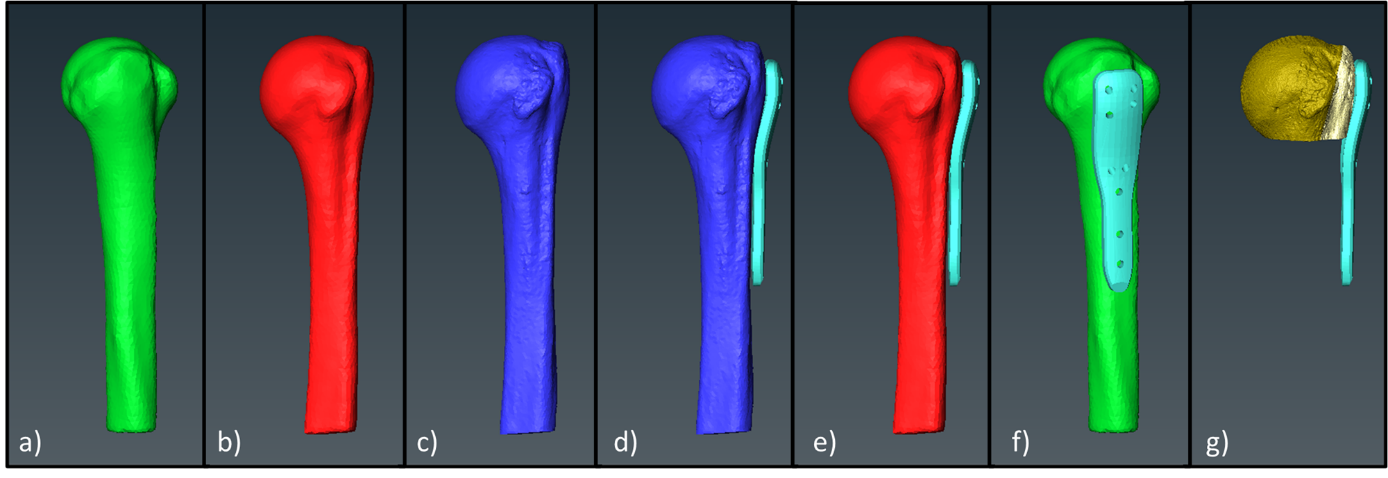


Figure S1 – Illustration of the steps for consistent implant positioning for the bone pair. The left humerus is shown in green (a) and the right side in red (b). The right bone was mirrored to mimic a left bone, which was then spatially registered to the left bone and both bone volumes are merged into one virtually combined bone volume (blue, c). The PHILOS plate was positioned on the combined bone (d). The defined plate positioning was applied to the left bone (e) and mirrored back to fit the right bone (f), ensuring the exact same positioning for both bones without interfering with the individual geometrical features. Similarly, the osteotomy planes (see Figure S2) were applied consistently on the left and right bones, resulting the separated head (g, gold) and greater tuberosity (g, beige) fragments.

# Specimen-specific 3D-printed guides – methodological details

Multi-purpose guides were designed and 3D-printed to navigate the experimental implementation of the planned procedures (Figure S2). The computer models of the guides were created based on cuboids, which were cropped by the planned two osteotomy planes. Similarly, the planned plate position was implemented, and the volumes of the bone and the plate were subtracted from the cuboids, creating a negative imprint. The screw configuration of the given configuration (original (OG) or enhanced (EH)) was automatically imported, and the corresponding screw trajectory was cut into the cuboids, creating cylindrical voids with the diameter identical to that of the commercial PHILOS screw guide. The mirroring procedure described above in Section 1 was used to create the guiding blocks for the pair, ensuring symmetry of all aspects except for the screw trajectories. The cuboids were further divided into four parts, which were attached via small connectors allowing assembly and disassembly during the instrumentation process. Two additional cylinders parallel to the anatomical humeral shaft axis were subtracted from the cuboids, facilitating standardized embedding procedure prior to mechanical testing. Similar features were used to enable exact mounting of the constructs into the biomechanical testing setup.


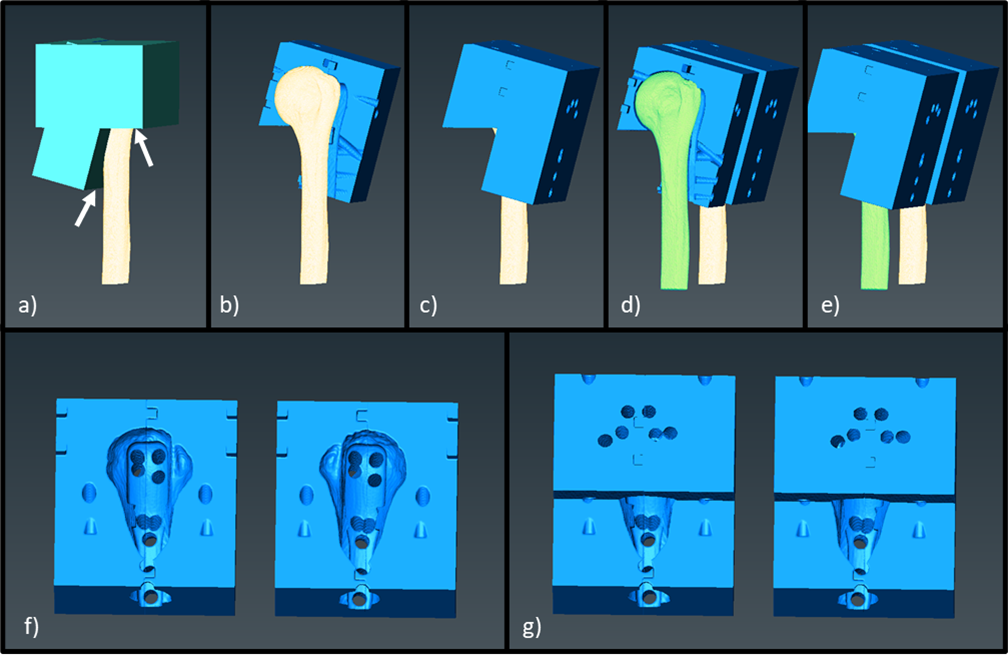


Figure S2 – Illustration of the computational workflow to create the specimen-specific guides. Two cuboids were positioned on the humeral head of the left bone with two sides (white arrows) defining the planned osteotomy planes (a). The bone, plate and screw holes were subtracted from the cuboids (b, c). The right bone (green) was mirrored and registered to the left bone and the subtraction procedure was repeated. The cuboids for the right bone were mirrored back (d, e), resulting in specific guides for both bones, ensuring consistency in implant positioning and osteotomy lines (f, g).

# Evaluation of instrumentation accuracy

The deviation of the achieved instrumentation from the planned configuration was evaluated on the post-operative CT scan (Figure S3) by measuring the screw angle differences in anterior-posterior and proximal-distal directions. Further, the tip-to-joint distance (TJD) was determined by measuring the distance from the screw tip to the projected point along the screw axis on the humeral head surface.

Screw orientation was achieved with a mean deviation of 0.04° (SD: 2.61°) in anterior-posterior direction and 0.36° (SD: 1.08°) in proximal-distal direction for the PHILOS group and a mean deviation of –0.07° (SD: 2.31°) in anterior-posterior direction and 0.33° (SD: 1.49°) in proximal-distal direction for the optimized group. When comparing the screw tip location with regards to the plate direction instead of the anatomical direction, mean screw tip deviation towards the left side of the plate was –1.99° (SD: 1.67°) for the PHILOS group and -1.41° (SD: 1.82°) for the optimized group. The much larger deviation for the screw tip placement in the anterior-posterior direction compared to the left-right direction of the plate shows that screw positioning error was mostly driven by a consistent error in the manufacturing process of the 3D printed plates and the CNC-tapped locking holes, rather than the anatomical properties of the bone. Furthermore, positioning of the screws in the proximal-distal direction was more accurate than positioning in the left-right plate direction. TJD in the achieved group was on average 5.72 mm (SD: 1.06 mm) with screw 2 (row A) being the most accurate (Mean ± SD: 6.01 mm ± 1.22) and screw 8 (row B) resulting in the least accurate TJD (Mean ± SD: 5.32 mm ± 0.78) compared to the targeted 6.00 mm. Nevertheless, the results were considered sufficiently accurate, given the 2 mm step increments in the commercially available screw lengths.


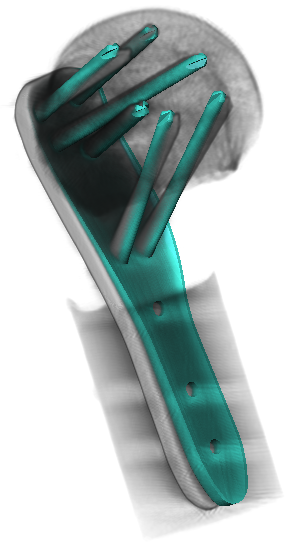


Figure S3 – CT image after instrumentation of an exemplary specimen (grayscale) with the planned instrumentation state overlayed (teal). Screw angles, screw length, plate position, bone fragment position and embedding alignment was evaluated and incorporated into the achieved models.
